# Applicability of RNA standards for evaluating RT-qPCR assays and platforms

**DOI:** 10.1186/1471-2164-12-118

**Published:** 2011-02-18

**Authors:** Alison S Devonshire, Ramnath Elaswarapu, Carole A Foy

**Affiliations:** 1 LGC Limited, Queens Road, Teddington, Middlesex, TW11 0LY, UK

## Abstract

The availability of diverse RT-qPCR assay formats and technologies hinder comparability of data between platforms. Reference standards to facilitate platform evaluation and comparability are needed. We have explored using universal RNA standards for comparing the performance of a novel qPCR platform (Fluidigm^® ^BioMark™) against the widely used ABI 7900HT system. Our results show that such standards may form part of a toolkit to evaluate the key performance characteristics of platforms.

## Background

Reverse transcription quantitative PCR (RT-qPCR) is rapidly becoming a valuable tool for mRNA biomarker quantification in clinical diagnostics. There has been a proliferation of RT-qPCR assay formats and platforms in recent years due to wider applications of this technology, coupled with improvements in sensitivity, specificity and accuracy of measurements of gene expression. However, there is one intrinsic limitation to the current qPCR platforms, namely lack of controls for cross-platform comparison. Although the manufacturers have developed platform-specific quality controls, they are often not adequate for cross-platform comparisons, particularly for the evaluation and standardization of transcriptomic data due to differences in protocols, data processing and analysis methods. Thus, development of universal RNA standards offers great potential in the validation of data obtained from different RT-qPCR methods. In the present investigation, we have compared the performance of Fluidigm^® ^BioMark™ Integrated microfluidic (henceforth referred to as BioMark) dynamic arrays with the widely used ABI 7900HT real-time PCR platform (henceforth called ABI 7900HT system) using generic RNA standards.

Pre-amplification of RNA or cDNA facilitates the investigation of a large number of genes when the starting material is limiting, such as with tissue biopsies and archival formalin-fixed paraffin-embedded (FFPE) samples [1,2]. Pre-amplification methods used generally include either linear amplification of RNA or exponential (PCR-based) amplification of cDNA [3-5]. However, concerns have been raised as to whether pre-amplification of samples by exponential amplification introduces bias in expression levels between genes [6]. For the BioMark microfluidic PCR system, each sample in the 48 × 48 dynamic array is distributed amongst 48 different reaction chambers, therefore pre-amplification is recommended for certain applications. However the limit of detection (LOD) of using pre-amplified *vs*. non-amplified cDNA samples, and its impact on the technical performance of the PCR array have not been fully characterized.

Exogenous RNA controls produced by *in vitro *transcription are ideal materials for investigating different RT-qPCR kits and methodologies [7]. Recently a panel of RNA controls have been developed for use in gene expression applications by the External RNA Controls Consortium (ERCC), an *ad hoc *group of 70 members from private, public and academic organizations led by the National Institute of Standards (NIST) [8,9]. It is hoped that standards developed from these sequences will aid in comparisons of gene expression data generated from various platforms such as microarray, RT-qPCR and next generation sequencing, and also provide quality control of gene expression measurements in the clinical laboratory [10]. Multigene biomarker measurements are at the forefront of a new class of medical devices using *in vitro *diagnostic multivariate assays, such as MammaPrint and Oncotype Dx in the area of breast cancer prognosis [11]. Since gene expression biomarkers typically encompass a range of transcript abundances and differential expression ratios, it is more appropriate to use multiple RNA standards as quality controls for standardizing such measurements, as opposed to a single transcript at a fixed concentration.

In the current study, we used a sub-set of the 96 ERCC RNA standards (Additional File 1) in order to characterize their performance on a nanofluidic PCR system, the BioMark 48 × 48 dynamic arrays, against a conventional qPCR platform, the ABI 7900HT system. We also investigated the impact of pre-amplification of cDNA samples on the linear range and precision of measurements by nanofluidic qPCR. Two prototype panels were constructed with selected RNA standards containing varying copy number within each panel, and varying ratios between them for mimicking non-differentially and differentially expressed mRNA biomarkers as represented in normal and disease states. The expression profile of the RNA standards was measured using both platforms and the accuracy and precision of their detection were compared.

## Results

### Linear range of dynamic arrays

One advantage of the BioMark arrays is the capability to analyse a large number of genes in a single sample. In order to facilitate this, up to ~ 2 μL of sample is loaded into each sample inlet of the chip and further distributed in the channels of the microfluidic chip as 48 separate 9 nL reactions using the integrated fluidic circuit (IFC). Thus the original sample is diluted more than 200-fold prior to the PCR reaction. In order to ensure that there are sufficient copies of target molecules in each reaction, Fluidigm^® ^recommends using either RNA samples that do not have a concentration lower than 250 ng total RNA/μL or that a pre-amplification stage is included, whereby the cDNA sample undergoes 14-18 cycles of amplification with a mix of up to 100 different primer pairs (Fluidigm Advanced Development Protocols 3, 5 and 8). In order to further investigate the requirement for pre-amplification, RNA standards were spiked into human total RNA at different concentrations (for sample composition, see Additional File 2) with the aim of mimicking a range of physiological abundances, from highly abundant mRNA transcripts (10^6 ^copies/ng total RNA; equivalent to 10^4 ^copies per cell) to transcripts only expressed in a sub-population of cells (1 copy/ng total RNA; equivalent to 0.01 copies per cell), based on the RNA content of a cell estimated as 26 pg [12]. A single RT reaction was performed for each RNA sample followed by 3 independent qPCR runs, with replicate assay measurements for each ERCC standard.

**Figure 1:**
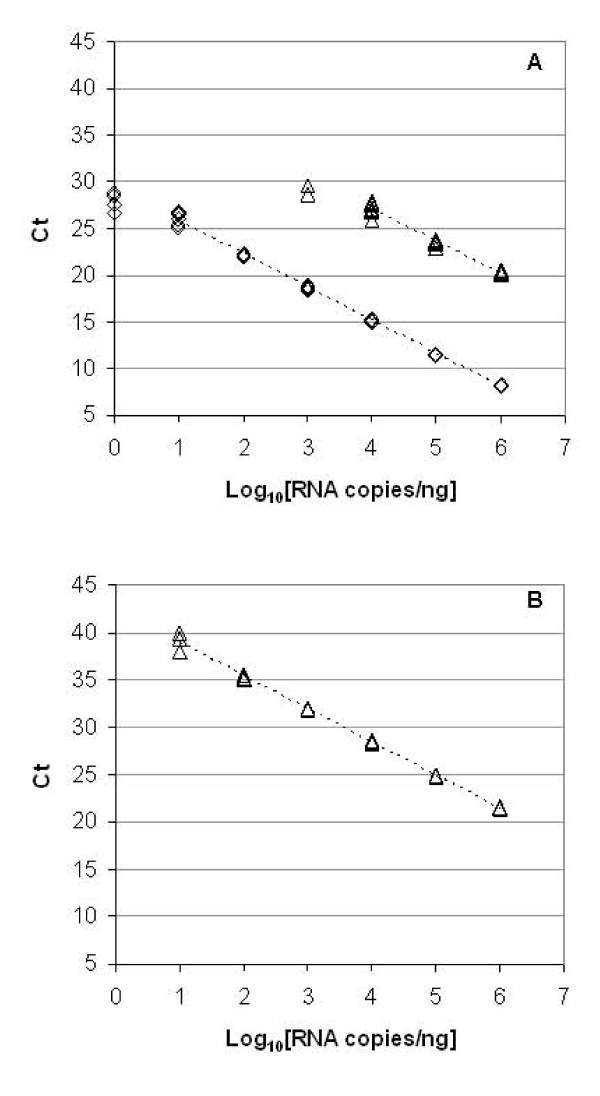
**Linear range of RT-qPCR platforms**. Example plot showing detection of an ERCC RNA standard (ERCC-42) across a range of transcript copy numbers from 1 to 10^6 ^copies per ng total RNA with cDNA (triangles) or pre-amplified cDNA (diamonds) as the template on (A) BioMark (B) ABI 7900HT system. Data-points are displayed as individual qPCR replicates. Dotted line indicates linear detection range.

Figure 1 compares the results of real-time PCR with cDNA samples (equivalent to 1 ng total RNA) or pre-amplified cDNA on the BioMark arrays with non-amplified cDNA using the ABI 7900HT system. The linear range in terms of transcript copy numbers for the pre-amplified cDNA samples on the BioMark arrays was similar to the non-amplified cDNA samples on the ABI 7900HT system, covering five orders of magnitude between 10 and 10^6 ^copies/ng total RNA. Although the linear range of both platforms was similar, the Ct values from BioMark were over 10 units lower than those observed for ABI 7900HT system. This could be due to the higher concentration of the template in the 9 nL nanofluidic reaction chambers of BioMark arrays compared to the standard 20 μL volume used on the ABI 7900HT system, such that the fluorescence output of the PCR reaction exceeds the threshold level at an earlier cycle (personal communication: A. Meliss, Fluidigm, September 2009). For the RT-PCRs performed with non-amplified cDNA samples on BioMark arrays, the linear detection range covered only two orders of magnitude between the transcript numbers of 10^4 ^and 10^6 ^copies/ng.

### LOD of dynamic arrays

**Figure 2:**
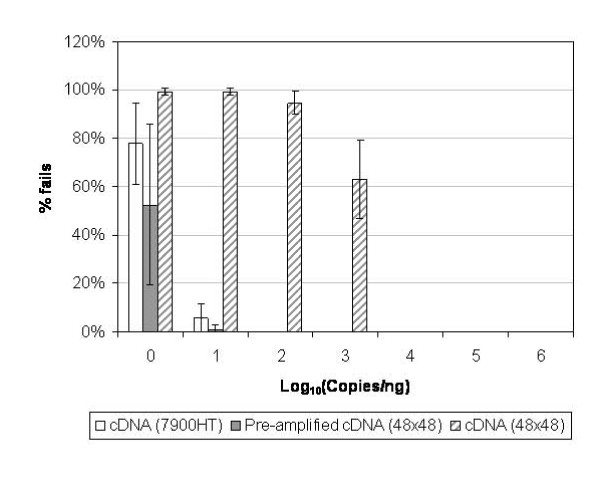
**Limit of detection of ERCC RNA standards**. Incidence of failed PCR reactions on ABI 7900HT system or BioMark using cDNA or pre-amplified cDNA as the template. Mean percentage of failed PCR reactions ± SD are displayed based on data from 8 different RNA standards from 3 independent qPCR experiments across a range from 1 to 10^6 ^copies ERCC standard per ng total RNA.

Unlike hybridization-based technologies such as microarrays, the LOD cannot be ascribed for RT-qPCR using a baseline for sample blanks, as a Ct value is not obtained for zero control samples. Therefore, the incidence of failed PCR reactions (undetermined Ct value) across the range of transcript abundances was also compared for conventional and nanofluidic PCR platforms with pre- or non-amplified cDNA as the template (Figure 2). As in Figure 1, a high percentage of PCR failures was observed at 1 copy/ng total RNA for both pre-amplified cDNA on the BioMark arrays and cDNA using the ABI 7900HT system. The rate of failures was slightly lower on the BioMark arrays as the projected template concentration per 9 nL reaction was 6 copies (assuming 100% efficiency of RT and pre-amplification), whilst it was only 1 RNA copy per ABI 7900HT reaction. For non-amplified cDNA, high reaction failure rates with BioMark arrays were observed below 10^3 ^copies/ng, which equates to 2 copies per 9 nL reaction.

### qPCR accuracy and precision

The accuracy and precision of qPCR detection across the above noted linear range was assessed by linear regression, comparing the slope and R^2 ^of the data from three independent dynamic arrays (Table 1). Pre-amplification of cDNA samples resulted in a significant improvement in the slope of the linear regression of copy number against Ct value (*p *< 0.05), with the mean slope within 6% of the ideal slope of 1. The pre-amplified cDNA samples demonstrated greater precision of the instrument over the linear detection range (Figure 1) than cDNA (*p *< 0.05) (Table 1). The accuracy and precision of pre-amplified cDNA detection on the BioMark arrays were comparable to those of the ABI 7900HT where non-amplified cDNA was used as the template (Table 1).

**Table 1:** Accuracy and precision of linear detection range of PCR platforms

	Slope			**R**^**2**^		
**Platform**	**Dynamic arrays**		**ABI 7900HT**	**Dynamic arrays**		**ABI 7900HT**

**Template**	**Pre-amplified** **cDNA**	**cDNA**	**cDNA**	**Pre-amplified** **cDNA**	**cDNA**	**cDNA**
						
**ERCC-**						

13	-1.06	-1.08	-1.06	0.999	0.993	0.997

42	-1.07	-1.11	-1.06	0.998	0.970	0.995

81	-1.02	-1.07	-1.04	0.998	0.976	0.998

84	-1.03	-1.07	-1.01	0.998	0.973	0.996

95	-1.07	-1.12	-1.08	0.997	0.975	0.998

99	-1.06	-1.06	-1.05	0.998	0.985	0.997

113	-1.06	-1.15	-1.04	0.998	0.971	0.997

171	-1.12	-1.13	-1.08	0.998	0.982	0.996

All	-1.06	-1.10	-1.05	0.999	0.978	0.997

Linear regression was performed with Ct values vs. log_2_(ERCC RNA copy number) for each PCR platform across the linear range marked in Figure 1. The mean slope and R^2 ^values from three independent qPCR experiments reflect the accuracy and precision of the detection of the 10-fold differences in copy numbers between samples.

**Figure 3:**
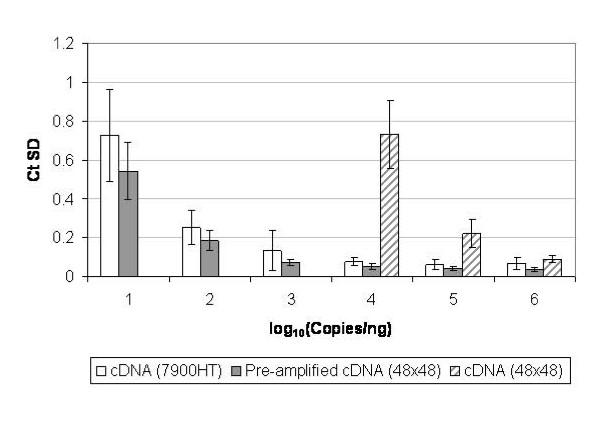
**Precision of real-time PCR platforms**. Within-run qPCR precision across a range of transcript abundance levels (copies per ng total RNA) are displayed for cDNA or pre-amplified cDNA quantified using ABI 7900HT system or BioMark. Mean variation (SD) of Ct values ± SD is displayed based on data from eight different RNA standards from three independent qPCR experiments.

Precision of qPCR detection as a function of transcript copy number for the two platforms is shown in Figure 3. For concentrations of RNA standards above 10^4 ^copies/ng (using cDNA with the ABI 7900HT system or pre-amplified cDNA for the BioMark arrays), and above 10^6 ^copies/ng (with non-amplified cDNA for the BioMark arrays), Ct standard deviation values are below 0.1 units, corresponding to less than 7% variation [13]. As RNA copy numbers decrease, variation between replicate qPCR measurements increases, with maximum average standard deviation values corresponding to 46% and 66% variation at 10 copies/ng for the BioMark arrays (pre-amplified cDNA) and ABI 7900HT system respectively (Figure 3).

### RNA biomarker panels

**Figure 4:**
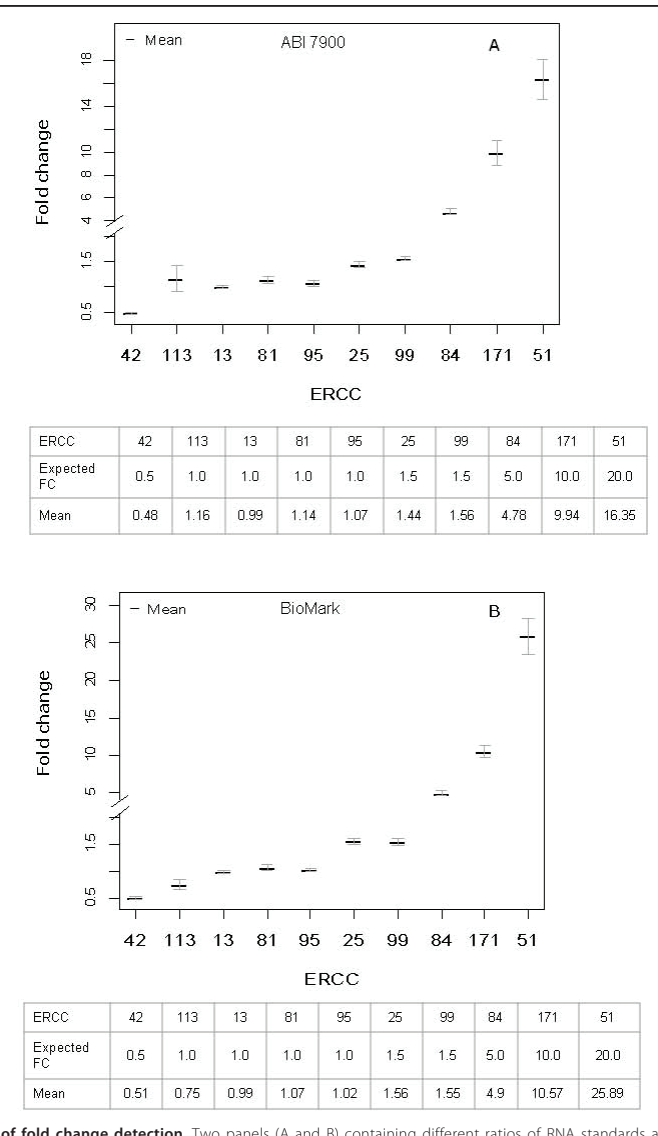
**Accuracy of fold change detection**. Two panels (A and B) containing different ratios of RNA standards across a range of copy numbers were quantified using (A) cDNA with the ABI 7900HT system or (B) pre-amplified cDNA with BioMark 48 × 48 dynamic arrays. Expected fold change values (see the tables below the Figures) are compared to the mean of measured fold changes from RT-qPCR reactions performed on six pairs of samples. The error bars represent standard error.

The accuracy of nanofluidic dynamic PCR for detection of multiple genetic biomarkers was further tested using two panels of RNA standards. With the aim of mimicking 'normal' and 'disease' states where some biomarkers are differentially expressed whilst others remain unchanged in their expression, standards were spiked at different ratios (1.0, 1.5, 2.0, 5.0, 10.0 and 20-fold differences) over a range of transcript copy numbers (Table 2). Three independent RT experiments, each containing two replicate RT reactions, were performed in order to investigate how technical noise associated with the whole RT-qPCR process impacts on the detection of differential or non-differential transcript expression levels. The resulting cDNA was quantified on the ABI 7900HT system or pre-amplified and measured on the BioMark. Fold change values were calculated using ΔCt values and the results of pair-wise comparison of the expression levels of each ERCC standard in the two panels displayed in Figure 4.

**Table 2:** Concentrations and ratios of ERCC RNA standards in simulated 'normal' and 'disease' panels

ERCC standard	Copies/ng total RNA		Ratio B/A
		
	Panel A	Panel B	
13	1 × 10^5^	1 × 10^5^	1.0

25	1 × 10^2^	1.5 × 10^2^	1.5

42	1 × 10^4^	5 × 10^3^	0.5

51	5 × 10^0^	1 × 10^2^	20.0

81	1 × 10^2^	1 × 10^2^	1.0

84	1 × 10^2^	5 × 10^2^	5.0

95	1 × 10^3^	1 × 10^3^	1.0

99	8 × 10^3^	1.2 × 10^4^	1.5

113	1 × 10^1^	1 × 10^1^	1.0

171	1 × 10^1^	1 × 10^2^	10.0

10 ERCC RNA standards were spiked in a background of Universal Human Reference RNA at different concentrations and ratios in order to create simulated 'normal' and 'disease' panels, A and B.

Overall, fold change estimation was found to be accurate for both ABI 7900HT system and BioMark arrays (Figures 4A and 4B), with the observed fold change values overlapping with the expected fold change measurements for all standards. For ERCC standards mimicking low abundance transcripts, the technical noise associated with the resulting fold change measurement was considerably greater than higher abundance RNA species. For example, a 20-fold increase in expression level at 5 copies/ng (ERCC-51) is associated with 16-fold and 10-fold difference in the minimum and maximum fold changes detected by the BioMark arrays and ABI 7900HT system respectively. For non-differential expression at low copy numbers (fold change = 1.0; ERCC-113), fold change measurements spanned a range of over 50% and 150% of the expected value on the BioMark and ABI 7900HT system respectively. At levels of abundance exceeding 100 copies/ng, mean fold change measurements were accurate to within 10% of expected values.

## Discussion

In this study we sought to demonstrate the utility of RNA standards for characterisation of a new platform, the BioMark, where PCR reactions are performed in volumes over a 1000-fold lower than on a conventional RT-qPCR instrument (ABI 7900HT system). The requirement for sample pre-amplification for this technology contrasts with standard two-step RT-qPCR approach, therefore the impact of this methodology was also evaluated. Dilutions of RNA standards across a wide physiological range demonstrated that the linear detection range of the BioMark arrays is similar to the ABI 7900HT real-time PCR system, when pre-amplified cDNA is used as the template (Figure 1). The precision of replicate measurements within the array also compared favourably to the intra-run standard deviation of Ct values for the ABI 7900HT system (Figure 3). At copy numbers mimicking medium to high abundance transcripts, the precision of the BioMark arrays is in a similar range to the minimum variation of ~ 0.1 units observed for another nanofluidic PCR array, the OpenArray format [14].

However, when non-amplified cDNA is quantified using the nanofluidic BioMark arrays, the linear range is severely limited, to only two orders of magnitude (Figure 1). At the lower detection limit of 10^4 ^RNA copies per reaction (Ct ~ 27), the variation between measurements increases significantly (Figure 3), whilst below this level of abundance, the rate of PCR failures increases rapidly (Figure 2). Pre-amplification of template cDNA using a preliminary PCR step of 14 cycles improves both the accuracy and precision of the transcript quantification using the dynamic arrays (Table 1). The improved detection of the 10-fold differences in RNA copy numbers between sample series (resulting in an average slope within 6% of expected value) also indicates that the pre-amplification process does not introduce bias into the detection of transcripts which cover a wide dynamic range.

Relative expression measurements are central to gene expression analysis by RT-qPCR and for determining whether a panel of biomarkers has predictive power for disease diagnosis and prognosis [15]. Therefore, we developed two panels of RNA standards in order to further investigate the accuracy of detecting gene expression ratios using the new type of PCR array compared to an established system. The standards were spiked at varying ratios between panels in order to obtain information on how well the methodologies can discriminate between differentially and non-differentially expressed candidate genes at different transcript abundance levels (Figure 4). Our results show good accuracy of observed *vs*. expected values for both platforms, which is in agreement with previous studies demonstrating good concordance of fold change measurements between the BioMark arrays and the ABI 7900HT system [16]. The precision of the fold change estimation varied according to the abundance of the transcripts, demonstrating increased variation in the observed values for lower concentrations of standards for both nanofluidic and standard real-time PCR approaches. This suggests that the sensitivity of the technique to correctly detect the expected fold change is reduced at low copy numbers (10 RNA copies or less per reaction on the ABI 7900HT system). Dixon et al. [14] also found that the sensitivity of the OpenArray platform was lower for Ct values corresponding to lower copy numbers, resulting in an increased number of false negative results. The increased variation in fold change detection at low copy numbers is likely to arise due to decreased efficiency at RT stage and increased stochastic variation in the PCR reaction for low target numbers [17].

We also found that both qPCR platforms were able to accurately detect a 1.5-fold change in mRNA expression, below the 2-fold cut-off which has been cited as a limit to the resolving power of conventional PCR, as it constitutes a difference of less than a single cycle [18]. The BioMark dynamic arrays were recently shown to be able to detect a 1.25-fold difference in DNA copy number by qPCR, with greater levels of precision achievable with the larger number of technical replicates possible with this high-throughput approach [19]. Since assay and sample loadings are in separate inlets on 48 × 48 dynamic arrays, it is possible to increase technical replication by using multiple assay inlets and/or multiple sample inlets. However, it should be noted that replication only at the assay level does not substitute for true sampling variation by the process of taking a sample from a population of molecules.

The use of gene-specific oligonucleotide standards for inter-run and cross-platform calibration has been demonstrated to improve the accuracy of class prediction based on panels of biomarkers [15]. Although ERCC RNA standards do not directly provide information on the performance of biomarker-specific assays, a panel of multiple standards, such as that used here provides a robust means of evaluating platform performance by minimizing confounding effects resulting from differences in assay performance due to individual primer and probe specificity. RNA standards could also serve as calibrator samples between experiments where different sets of potential biomarkers genes are investigated, as well as in the context of a diagnostic assay where the expression of the same panel of genes is quantified. In addition to target gene normalization using a reference gene or panel of reference genes [20], normalization to an ERCC RNA standard or multiple RNA standards may be a useful control for elucidating technical variation due to RT and qPCR steps [21].

## Conclusions

We conclude that universal RNA standards can provide robust information on the performance characteristics of different RT-qPCR platforms and methodologies. The results obtained using panels of multiple RNA standards indicate that the linear detection range, precision and accuracy of nanofluidic BioMark dynamic arrays are similar to those of an established real-time PCR instrument, the ABI 7900HT system, when pre-amplified cDNA is used as the template. The standards also provide reference values for the range of transcript abundance over which it would be possible to measure non-amplified cDNA on the nanofluidic BioMark high-throughput arrays. Carefully constructed panels of ERCC RNA standards have the potential to act as benchmarks for the calibration and interpretation of biomarker measurements in drug discovery and clinical diagnostics. Further evaluation of these standards is required for potential incorporation into a 'quality metrics' toolkit for assessing their suitability for cross-platform comparisons.

## Methods

### Preparation of *in vitro *transcribed RNA and samples

*In vitro *transcribed ERCC RNA standards were produced from ERCC plasmid DNA (courtesy of Dr. Marc Salit, NIST, USA). Plasmid DNA from standards ERCC-13, 25, 42, 51, 81, 84, 95, 99, 113 and 171 was cleaved into a single linear molecule using *Bam*HI restriction endonuclease (New England Biolabs, UK). 500 ng of plasmid DNA was used for each sample and digested by adding 40 U of *Bam*HI enzyme in NEB3 buffer provided by the manufacturer. The digestion mixture was incubated at 37°C for 2 hours followed by purification using QiaQuick PCR purification kit with an elution volume of 32 μl. *In vitro *transcription was carried out with 8 μl digested plasmid DNA using MEGAscript^® ^T7 Kit (Applied Biosystems/Ambion, UK) followed by DN*ase *treatment and clean-up using RNeasy columns (Qiagen, UK). RNA concentration and insert sizes were estimated using the Nanodrop 1000 spectrophotometer (Thermo Scientific, UK) and 2100 Bioanalyzer (Agilent Technologies, USA) respectively. RNA standards were diluted in nuclease free-water and spiked into Universal Human Reference RNA (Stratagene, UK) (final concentration 100 ng/μl). For experiments investigating the linear range of platform detection, standards were spiked at 10-fold intervals between 1 and 10^6 ^copies/ng total RNA (Additional File 2). For the simulated 'normal' and 'disease' panels, standards were spiked at various copy numbers and ratios (Table 2).

### Reverse transcription and pre-amplification of cDNA

RNA samples were reverse-transcribed using the TaqMan^® ^Reverse Transcription Reagents kit (Applied Biosystems, UK) in 40 μL reactions containing 400 ng total RNA and oligo(dT) primers according to manufacturer's instructions. cDNA samples were diluted to a concentration of 0.5 ng/μL (total RNA equivalent) with nuclease-free water. For experiments investigating the linear range of platform detection (Figures 1, 2, 3), a single RT reaction was performed for each RNA sample whilst for the simulated 'normal' and 'disease' panels (Figure 4), 6 replicate RT reactions were performed. A single aliquot of each cDNA sample, equivalent to 12.5 ng RNA, was pre-amplified with assays corresponding to all 10 standards in a 25 μL volume reaction using TaqMan^® ^PreAmp Mastermix (Applied Biosystems, UK) according to manufacturer's protocol. Following pre-amplification, the samples were diluted 1:5 (v/v) in TE buffer, pH 8.0.

### Real-time PCR

Further information on sample preparation and real-time PCR validation complying with the Minimum Information for Publication of Quantitative Real-Time PCR Experiments (MIQE) guidelines [22] is available in Additional Files 2 and 3 (MIQE Additional Information and Checklist).

Custom-designed primers and TaqMan^® ^FAM-TAMRA probes for each ERCC standard (Additional File 1) were supplied by Applied Biosystems and a 20 × assay mix was prepared containing 18 μM primer and 5 μM probe (final concentration 900 nM primer and 250 nM probe). qPCR assays were tested initially using a serial dilution of ERCC cDNA and PCR efficiencies calculated (see Additional Data Table 2: MIQE Additional information). All 10 assays were found to have PCR efficiencies of greater than 86%.

BioMark arrays were prepared according to the manufacturer's instructions. TaqMan^® ^assays were diluted 1:1 (v/v) with DA Assay Loading Reagent (Fluidigm^®^) and 5 μL was added to each assay inlet of the array. Also, 5 μL reaction mix was prepared by mixing 2 × TaqMan^® ^Universal Mastermix (Applied Biosystems), DA Sample Loading Reagent and nuclease-free water containing 2 μL of cDNA or pre-amplified cDNA. The samples were loaded into each sample inlet as per manufacturer's recommendations. Following loading of the assays and samples into the chip by the IFC controller, PCR was performed with the following reactions conditions: 50°C for 2 minutes, 95°C for 10 minutes, followed by 45 cycles of 95°C for 15 seconds and 60°C for 60 seconds. Data was processed by automatic global threshold setting with the same threshold value for all assays and linear baseline correction using BioMark Real-time PCR Analysis software (version 2.1.1). The quality threshold was set at the default setting of 0.65. For experiments investigating the linear range of platform detection (Figures 1, 2, 3), 8 qPCR reactions consisting of 4 assay inlet and 2 sample inlet replicates were performed for each cDNA or pre-amplified cDNA sample. For the simulated 'normal' and 'disease' panels (Figure 4), 12 qPCR reactions consisting of 4 assay inlet and 3 sample inlet replicates were performed for each cDNA sample.

Conventional real-time PCR was performed using ABI 7900HT system in 20 μL reaction volumes containing TaqMan^® ^Universal PCR Master Mix and 2 μL of respective cDNA in optical 96-well plates (Applied Biosystems). Cycling conditions were as those used for the BioMark arrays. Triplicate qPCR reactions were performed for each cDNA sample for all experiments. The threshold fluorescence level was set manually for each plate using SDS software version 2.3 (Applied Biosystems). Following export of Cycle threshold (Ct) data, further data analysis for both platforms was performed in Microsoft^® ^Excel 2003. Comparison of slope and R^2 ^values between pre-amplified and non-amplified cDNA, as a template on the BioMark arrays, was performed as paired *t*-test in Microsoft^® ^Excel 2003.

## List of abbreviations

ERCC: External RNA Controls Consortium; RT-qPCR: Reverse Transcription Quantitative PCR; LOD: limit of detection; FFPE: formalin-fixed paraffin-embedded; IFC: integrated fluidic circuit; RT: Reverse Transcription; PCR: polymerase chain reaction.

## Authors' contributions

AD performed RT-qPCR experiments, participated in the design of the study, performed data analysis and drafted the manuscript. RE participated in the design of the study and helped to draft the manuscript. CF conceived of the study, and participated in its design and coordination and helped to draft the manuscript. All authors read and approved the final manuscript.

## Additional files

The following additional are available with the online version of this paper. Additional data file 1 is a table detailing the primer and probe sequences used for qPCR assays. Additional files 2 and 3 are additional data and a checklist in compliance with the MIQE (Minimum Information for Publication of Quantitative Real-Time PCR Experiments) guidelines.

## Supplementary Material

Additional file 1 **Taqman assays for ERCC RNA standards**. Microsoft Word file detailing the sequences of primers and probes used for qPCR assays. Click here for file

Additional file 2 **MIQE Additional Information**. Microsoft Excel file containing further information on RNA preparation, purity, PCR efficiency and negative control data complying with the MIQE guidelines. Click here for file

Additional file 3 **MIQE Checklist**. Checklist in Microsoft Word format detailing information complying with MIQE guidelines. Click here for file

## References

[B1] Vermeulen J Derveaux S Lefever S De Smet E De Preter K Yigit N De Paepe A Pattyn F Speleman F Vandesompele J RNA pre-amplification enables large-scale RT-qPCR gene-expression studies on limiting sample amounts BMC Res Notes 2009 2 235 10.1186/1756-0500-2-235 19930725 PMC2789097

[B2] Li J Smyth P Cahill S Denning K Flavin R Aherne S Pirotta M Guenther SM O'Leary JJ Sheils O Improved RNA quality and TaqMan Pre-amplification method (PreAmp) to enhance expression analysis from formalin fixed paraffin embedded (FFPE) materials BMC Biotechnol 2008 8 10 10.1186/1472-6750-8-10 18254955 PMC2259333

[B3] Eberwine J Yeh H Miyashiro K Cao Y Nair S Finnell R Zettel M Coleman P Analysis of gene expression in single live neurons Proc Natl Acad Sci USA 1992 89 3010 3014 10.1073/pnas.89.7.3010 1557406 PMC48793

[B4] Kurn N Chen P Heath JD Kopf-Sill A Stephens KM Wang S Novel isothermal, linear nucleic acid amplification systems for highly multiplexed applications Clin Chem 2005 51 1973 1981 10.1373/clinchem.2005.053694 16123149

[B5] Iscove NN Barbara M Gu M Gibson M Modi C Winegarden N Representation is faithfully preserved in global cDNA amplified exponentially from sub-picogram quantities of mRNA Nat Biotechnol 2002 20 940 943 10.1038/nbt729 12172558

[B6] Ginsberg SD RNA amplification strategies for small sample populations Methods 2005 37 229 237 10.1016/j.ymeth.2005.09.003 16308152

[B7] Levesque-Sergerie JP Duquette M Thibault C Delbecchi L Bissonnette N Detection limits of several commercial reverse transcriptase enzymes: impact on the low- and high-abundance transcript levels assessed by quantitative RT-PCR BMC Mol Biol 2007 8 93 10.1186/1471-2199-8-93 17953766 PMC2151766

[B8] External RNA Controls Consortium Proposed methods for testing and selecting the ERCC external RNA controls BMC Genomics 2005 6 150 10.1186/1471-2164-6-150 16266432 PMC1325234

[B9] Baker SC Bauer SR Beyer RP Brenton JD Bromley B Burrill J Causton H Conley MP Elespuru R Fero M Foy C Fuscoe J Gao X Gerhold DL Gilles P Goodsaid F Guo X Hackett J Hockett RD Ikonomi P Irizarry RA Kawasaki ES Kaysser-Kranich T Kerr K Kiser G Koch WH Lee KY Liu C Liu ZL Lucas A The External RNA Controls Consortium: a progress report Nat Methods 2005 2 731 734 10.1038/nmeth1005-731 16179916

[B10] Cronin M Ghosh K Sistare F Quackenbush J Vilker V O'Connell C Universal RNA Reference Materials for Gene Expression Clin Chem 2004 50 1464 1471 10.1373/clinchem.2004.035675 15155546

[B11] Ross JS Multigene classifiers, prognostic factors, and predictors of breast cancer clinical outcome Adv Anat Pathol 2009 16 204 215 10.1097/PAP.0b013e3181a9d4bf 19546609

[B12] Clinical and Laboratory Standards Institute Use of External RNA Controls in Gene Expression Assays 2010 Approved Guideline 26 No. 29, MM16-A.

[B13] Stahlberg A Hakansson J Xian X Semb H Kubista M Properties of the reverse transcription reaction in mRNA quantification Clin Chem 2004 50 509 15 10.1373/clinchem.2003.026161 14726469

[B14] Dixon JM Lubomirski M Amaratunga D Morrison TB Brenan CJ Ilyin SE Nanoliter high-throughput RT-qPCR: a statistical analysis and assessment Biotechniques 2009 46 ii viii 10.2144/000112746 19480642

[B15] Vermeulen J Pattyn F De Preter K Vercruysse L Derveaux S Mestdagh P Lefever S Hellemans J Speleman F Vandesompele J External oligonucleotide standards enable cross laboratory comparison and exchange of real-time quantitative PCR data Nucleic Acids Res 2009 37 e138 10.1093/nar/gkp721 19734345 PMC2790878

[B16] Spurgeon SL Jones RC Ramakrishnan R High throughput gene expression measurement with real time PCR in a microfluidic dynamic array PLoS One 2008 3 e1662 10.1371/journal.pone.0001662 18301740 PMC2244704

[B17] Bustin SA Nolan T Pitfalls of quantitative real-time reverse-transcription polymerase chain reaction J Biomol Tech 2004 15 155 166 15331581 PMC2291693

[B18] Lo YM Lun FM Chan KC Tsui NB Chong KC Lau TK Leung TY Zee BC Cantor CR Chiu RW Digital PCR for the molecular detection of fetal chromosomal aneuploidy Proc Natl Acad Sci USA 2007 104 13116 13121 10.1073/pnas.0705765104 17664418 PMC1934923

[B19] Weaver S Dube S Mir A Qin J Sun G Ramakrishnan R Jones RC Livak KJ Taking qPCR to a higher level: Analysis of CNV reveals the power of high throughput qPCR to enhance quantitative resolution Methods 2010 50 271 6 10.1016/j.ymeth.2010.01.003 20079846

[B20] Vandesompele J De Preter K Pattyn F Poppe B Van Roy N De Paepe A Speleman F Accurate normalization of real-time quantitative RT-PCR data by geometric averaging of multiple internal control genes Genome Biol 2002 3 RESEARCH0034 10.1186/gb-2002-3-7-research0034 12184808 PMC126239

[B21] Gilsbach R Kouta M Bonisch H Bruss M Comparison of *in vitro *and *in vivo *reference genes for internal standardization of real-time PCR data Biotechniques 2006 40 173 177 10.2144/000112052 16526406

[B22] Bustin SA Benes V Garson JA Hellemans J Huggett J Kubista M Mueller R Nolan T Pfaffl MW Shipley GL Vandesompele J Wittwer CT The MIQE Guidelines: Minimum Information for Publication of Quantitative Real-Time PCR Experiments Clin Chem 2009 55 611 622 10.1373/clinchem.2008.112797 19246619

