# Supplementary material for: Applicability of RNA standards for evaluating RT-qPCR assays and platforms
Source: BMC Genomics. 2011 Feb 18;12:118. doi: 10.1186/1471-2164-12-118 (PMC3052187; doi:10.1186/1471-2164-12-118)
Supplement: Additional file 1 — Taqman assays for ERCC RNA standards. Microsoft Word file detailing the sequences of primers and probes used for qPCR assays. [file 1471-2164-12-118-S1.DOC]

**Supplementary Table 1: ABI Taqman assays for ERCC RNA standards**

| **ERCC #** | **Forward primer** | **Reverse primer** | **Taqman® probe** | **Amplicon size (bp)** |
| --- | --- | --- | --- | --- |
| **13** | CGGACATGGTGTTGGTCAAG | TTGTTGGGCGGACCGTAA | TGCATGAGGACCCGCAAATTCCTC | 66 |
| **25** | CGGTCGTGAACTGCTATAGGA | GGTAGTTTCGCTGGTTCGTT | AGCCTGATACGAGCGCACAACA | 67 |
| **42** | AGAGAGCTTTTGGCAATCCT | TCATTTGCTAAGGCAGTTAAAGA | TCACCAGTTCCCATGAATGTTCCAC | 73 |
| **51** | TTATGTCCCATTGGCTATTCCTTT | TCTGGGTTTTGCTAATCAAAATCA | ATCTCTGGCCCTGGAGCTATAACAACAACC | 80 |
| **81** | TGACGGCTTCAACGTTTTCA | TCTGATGTACCAGCGTGCAACT | TTTCCGCAGGTGGCGACCCTC | 65 |
| **84** | TGGATAAGCGAGGTCAGTCAAG | ATGCAGGCAAACGATCTACGT | ATTCGTTGCCTCCGGGTCC | 65 |
| **95** | GAGCGTTTTTATGCAGTTCATCTTT | GGATAAGATTGTTGAGTGGGCTTT | ACCTCATCCCACAAAGCCGCTTTCTT | 77 |
| **99** | TCGTCCATCCCTCAAGAGAGA | CGCAATCGCGTGTGAATG | CATGGAAAGAGCTCGACAAAATTTACTC | 71 |
| **113** | GCGACACCAACATCGTTACG | CCGCGCGTGAGCACTT | ACACACCGGACGCTTGGATCAGTG | 65 |
| **171** | TTAGTTTCGTGGCGGGATTT | CACGAATCGCACGGATGTT | AGGAAAACTGCGACTGTTCTTTAACC | 67 |

**Supplementary Table 2: RNA standard sequence information**

| **ERCC standard id.** | **GenBank accession** | **Length**  **(excl. polyA tail)** | **GC content (%)** |
| --- | --- | --- | --- |
| **ERCC-00013-01** | EF011062 | 784 | 42.8 |
| **ERCC-00025-01** | DQ883689 | 1970 | 50.8 |
| **ERCC-00042-01** | DQ516783 | 999 | 39.2 |
| **ERCC-00051-01** | DQ516740 | 250 | 37.6 |
| **ERCC-00081-02** | DQ854991 | 509 | 48.7 |
| **ERCC-00084-01** | DQ883682 | 970 | 50.3 |
| **ERCC-00095-01** | DQ516759 | 495 | 37.2 |
| **ERCC-00099-01** | DQ875387 | 1324 | 41.3 |
| **ERCC-00113-01** | DQ883663 | 820 | 50.7 |
| **ERCC-00171-01** | DQ854994 | 481 | 47.7 |
