# Supplementary material for: Applicability of RNA standards for evaluating RT-qPCR assays and platforms
Source: BMC Genomics. 2011 Feb 18;12:118. doi: 10.1186/1471-2164-12-118 (PMC3052187; doi:10.1186/1471-2164-12-118)
Supplement: Additional file 3 — MIQE Checklist. Checklist in Microsoft Word format detailing information complying with MIQE guidelines. [file 1471-2164-12-118-S3.DOCX]

| **ITEM TO CHECK** | **IMPORTANCE** | **CHECKLIST** | **Comment** |
| --- | --- | --- | --- |
| **EXPERIMENTAL DESIGN** |  |  |  |
| Definition of experimental and control groups | **E** | **✓** |  |
| Number within each group | **E** | **✓** |  |
| Assay carried out by core lab or investigator's lab? | D | **✓** | Investigator’s Lab. |
| Acknowledgement of authors' contributions | D | **✓** |  |
| **SAMPLE** |  |  |  |
| Description | **E** | **✓** |  |
| Volume/mass of sample processed | D | N/A |  |
| Microdissection or macrodissection | **E** | N/A |  |
| Processing procedure | **E** | **✓** |  |
| If frozen - how and how quickly? | **E** | N/A |  |
| If fixed - with what, how quickly? | **E** | N/A |  |
| Sample storage conditions and duration (especially for FFPE samples) | **E** | **✓** | IVT RNA, UHRR and experimental samples stored at -80°C.  Aliquots of all samples of ERCC standards in UHRR background made to avoid freeze-thaw. |
| **NUCLEIC ACID EXTRACTION** |  |  |  |
| Procedure and/or instrumentation | **E** | **✓** |  |
| Name of kit and details of any modifications | **E** | **✓** |  |
| Source of additional reagents used | D | N/A |  |
| Details of DNase or RNAse treatment | **E** | **✓** |  |
| Contamination assessment (DNA or RNA) | **E** | **✓** | IVT RNA assayed for contaminating plasmid DNA by qPCR assays using RT minus sample. |
| Nucleic acid quantification | **E** | **✓** |  |
| Instrument and method | **E** | **✓** |  |
| Purity (A260/A280) | D | **✓** | See ‘RNA preparation’ in Additional Data File 2.. |
| Yield | D | **✓** | See ‘RNA preparation’ in Additional Data File 2.. |
| RNA integrity method/instrument | **E** | **✓** |  |
| RIN/RQI or Cq of 3' and 5' transcripts | **E** | **✓** | See ‘RNA preparation’ in Additional Data File 2. |
| Electrophoresis traces | D |  |  |
| Inhibition testing (Cq dilutions, spike or other) | **E** | N/A | ERCCs are spike-ins. |
| **REVERSE TRANSCRIPTION** |  |  |  |
| Complete reaction conditions | **E** | **✓** |  |
| Amount of RNA and reaction volume | **E** | **✓** |  |
| Priming oligonucleotide (if using GSP) and concentration | **E** | **✓** | As manufacturer’s instructions. |
| Reverse transcriptase and concentration | **E** | **✓** | As manufacturer’s instructions. |
| Temperature and time | **E** | **✓** | As manufacturer’s instructions. |
| Manufacturer of reagents and catalogue numbers | D | **✓** |  |
| Cqs with and without RT | D* | **✓** | See ‘Purity Testing IVT RNA’ in Additional Data File 2. |
| Storage conditions of cDNA | D | **✓** | -20°C. |
| **qPCR TARGET INFORMATION** |  |  |  |
| If multiplex, efficiency and LOD of each assay. | **E** | N/A |  |
| Sequence accession number | **E** | **✓** | Additional Data File 1 |
| Location of amplicon | D |  |  |
| Amplicon length | **E** | **✓** | Additional Data File 1 |
| *In silico* specificity screen (BLAST, etc) | **E** | **✓** | No significant cross-reactivity with human transcripts (also see RT- data, Additional Data File 2). |
| Pseudogenes, retropseudogenes or other homologs? | D | N/A |  |
| Sequence alignment | D |  |  |
| Secondary structure analysis of amplicon | D |  |  |
| Location of each primer by exon or intron (if applicable) | **E** | N/A |  |
| What splice variants are targeted? | **E** | N/A |  |
| **qPCR OLIGONUCLEOTIDES** |  |  |  |
| Primer sequences | **E** | **✓** | Additional Data File 1 |
| RTPrimerDB Identification Number | D | N/A |  |
| Probe sequences | D** | **✓** | Additional Data File 1 |
| Location and identity of any modifications | **E** | **✓** | FAM-TAMRA probes. |
| Manufacturer of oligonucleotides | D | **✓** |  |
| Purification method | D | **✓** | Primers: HSP  Probes: HPLC |
| **qPCR PROTOCOL** |  |  |  |
| Complete reaction conditions | **E** | **✓** |  |
| Reaction volume and amount of cDNA/DNA | **E** | **✓** |  |
| Primer, (probe), Mg++ and dNTP concentrations | **E** | **✓** | Mg++ and dNTP not available from qPCR mastermix manufacturer. |
| Polymerase identity and concentration | **E** | **✓** |  |
| Buffer/kit identity and manufacturer | **E** | **✓** |  |
| Exact chemical constitution of the buffer | D | N/A | Not available (as above). |
| Additives (SYBR Green I, DMSO, etc.) | **E** | N/A |  |
| Manufacturer of plates/tubes and catalog number | D | **✓** |  |
| Complete thermocycling parameters | **E** | **✓** |  |
| Reaction setup (manual/robotic) | D | **✓** | Manual. |
| Manufacturer of qPCR instrument | **E** | **✓** |  |
| **qPCR VALIDATION** |  |  |  |
| Evidence of optimisation (from gradients) | D |  |  |
| Specificity (gel, sequence, melt, or digest) | **E** | N/A | Taqman assays |
| For SYBR Green I, Cq of the NTC | **E** | N/A |  |
| Standard curves with slope and y-intercept | **E** | **✓** | See ‘PCR efficiency’ in Additional Data File 2. |
| PCR efficiency calculated from slope | **E** | **✓** |  |
| Confidence interval for PCR efficiency or standard error | D |  |  |
| r2 of standard curve | **E** | **✓** |  |
| Linear dynamic range | **E** | **✓** | Figures 1 and 2 |
| Cq variation at lower limit | **E** | **✓** | Figure 3 |
| Confidence intervals throughout range | D |  |  |
| Evidence for limit of detection | **E** | **✓** | Figure 2 |
| If multiplex, efficiency and LOD of each assay. | **E** | N/A |  |
| **DATA ANALYSIS** |  |  |  |
| qPCR analysis program (source, version) | **E** | **✓** |  |
| Cq method determination | **E** | **✓** |  |
| Outlier identification and disposition | **E** | N/A |  |
| Results of NTCs | **E** | **✓** | See Additional Data File 2. |
| Justification of number and choice of reference genes | **E** | N/A |  |
| Description of normalisation method | **E** | N/A |  |
| Number and concordance of biological replicates | D | N/A |  |
| Number and stage (RT or qPCR) of technical replicates | **E** | **✓** |  |
| Repeatability (intra-assay variation) | E | **✓** | Figure 3 |
| Reproducibility (inter-assay variation, %CV) | D |  |  |
| Power analysis | D |  |  |
| Statistical methods for result significance | **E** | **✓** |  |
| Software (source, version) | E | **✓** |  |
| Cq or raw data submission using RDML | **D** |  |  |
|  |  |  |  |
| **Table 1.** MIQE checklist for authors, reviewers and editors. All essential information (E) must be submitted with the manuscript. Desirable | | | |
| information (D) should be submitted if available. If using primers obtained from RTPrimerDB, information on qPCR target, oligonucleotides, | | | |
| protocols and validation is available from that source. |  |  |  |
|  |  |  |  |
| *: Assessing the absence of DNA using a no RT assay is essential when first extracting RNA. Once the sample has been validated as | | | |
| RDNA-free, inclusion of a no-RT control is desirable, but no longer essential. | | |  |
|  |  |  |  |
| **: Disclosure of the probe sequence is highly desirable and strongly encouraged. However, since not all commercial pre-designed assay | | | |
| vendors provide this information, it cannot be an essential requirement. Use of such assays is advised against. | | | |
|  |  |  |  |
|  |  |  |  |
